# Supplementary material for: Association between oral microbiome and seven types of cancers in East Asian population: a two-sample Mendelian randomization analysis
Source: Front Mol Biosci. 2023 Nov 21;10:1327893. doi: 10.3389/fmolb.2023.1327893 (PMC10702768; doi:10.3389/fmolb.2023.1327893)
Supplement: Supplementary file 1 [file DataSheet1.ZIP › Supplementary Materials/MR plots for tongue/tongue═╝/Gastric cancer/pheno.1215_to_gastric cancer_leave_one_out.pdf]

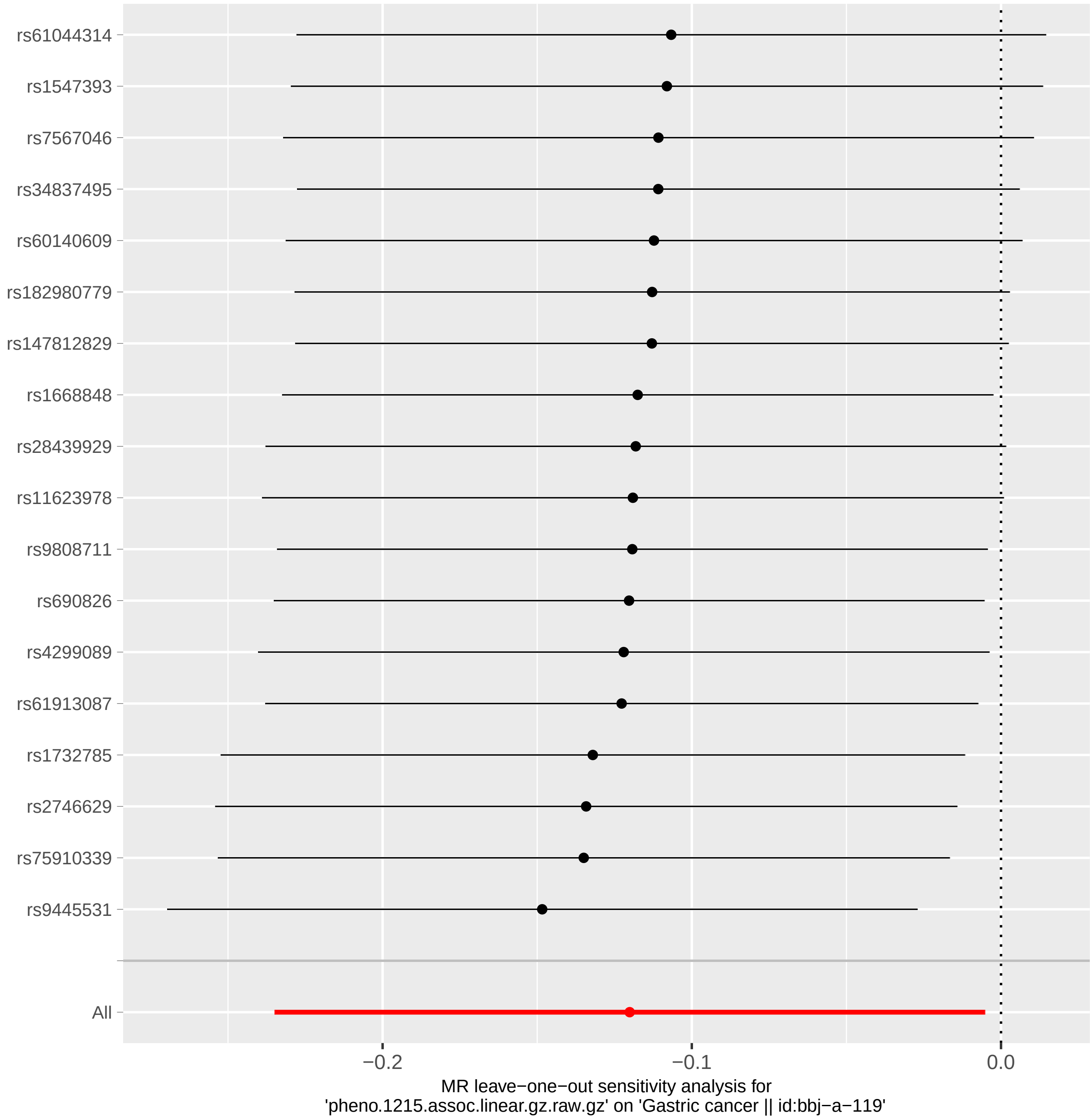
rs61044314
rs1547393
rs7567046
rs34837495
rs60140609
rs182980779
rs147812829
rs1668848
rs28439929
rs11623978
rs9808711
rs690826
rs4299089
rs61913087
rs1732785
rs2746629
rs75910339
rs9445531
All
−0.2
−0.1
0.0
MR leave−one−out sensitivity analysis for
'pheno.1215.assoc.linear.gz.raw.gz' on 'Gastric cancer || id:bbj−a−119'
